# The Opportunity of the Medical Profession

**Published:** 1917-06

**Authors:** 


### The Opportunity of the Medical Profession.

The worst problem with which the medical profession of the whole civilized world has had to contend, for many years, is excess of its own numbers. Other problems may seem worse because they involve departures from ideals of a higher order than dollars and comfortable living but there is no getting away from the fundamental importance of sound economics. Moreover, in the aggregate, disregarding individual instances, every higher ethical problem can be reduced to the economic basis. Why do quacks exist? Why do surgeons and others split fees surreptitiously? Why do doctors standing high in society and the church, do abortions? Why do doctors steal cases? Why do they go to the limits of dishonorable procedures especially for the sake of attending cases from which they will not receive a cent directly? Why are many men incompetent and unprovided with needed books and instruments? Why is there constant friction between the medical and pharmacal professions over counter prescribing and office dispensing? Is there any higher ethical problem which, barring individual crookedness, can not be explained by the fundamental fact that there are more doctors than can find reasonably profitable employment?

Never in the recent history of medicine has there been a time when the prospects of comparatively rapid relief of this fundamental economic evil have been so encouraging. When we pause to consider the reasons, it seems cold blooded and hard hearted to speak thus but, after all, no harm can come from looking at the bright side, especially as with, one possible exception, the relief of the economic evil has been forced on the medical profession from without, while the responsibility of initiating the sole method of relief that has arisen within the profession, rests on men who were at their prime 25-30 years ago, who are mostly dead or so far advanced in age as no longer to be in active professional life, who offered many plausible excuses for their views and received the support of most thinking persons. This factor of intra-professional relief, indeed, required for its establishment, the approval of the public through their legislators and executives. It has been assailed time and again but, with transient exceptions, has always been approved by the public and has been progressively increased in the depth and breadth of its influence. It is scarcely necessary to explain that it consisted in the cruel edict that a man who desired to enter the medical profession should have more than the education of a laborer and more technical training than a nurse.

The only other important factor in rendering professional adjustment to the law of supply and demand possible has been war and preparation for war. No one regrets this more than the writer but it would be foolish to deny its bearing on the economic and hence on ethical problems. There are no accurate statistics of the toll of life among European physicians, 400 British physicians are said to have been killed in the Somme campaign alone. It might seem that the present actual shortage of medical men in Europe was a transient condition but it must be remembered that, in war, about 7 medical officers are required per 1000 of healthy male adults who, in normal times, would require perhaps a fifth of the service of 1. In our country, the proposed increase of troops to 2 million, will require the more or less permanent withdrawal of at least 4 physicians per 1000, without actual hostilities, and at least 5000 physicians will be taken from the services of the civil population, beyond those already engaged by the military services. At present, the government wants about four times this estimate, available for orders.

Meantime, the additions to our numbers are only slightly more than are necessary to compensate for deaths on an average basis, less than would be required to maintain the ratio of physicians to population if the latter increases at its former rate, even without immigration. It must also be remembered that the medical profession is not normally distributed as to age but is top-heavy in this particular. The maximum actual, and still higher relative, proportion of physicians by age groups to the total, is of those who graduated 10-20 years ago. Within a comparatively few years, by the natural course of events, the medical profession will be rapidly depleted. There is no immediate danger of there being a scarcity of physicians for disease incidence is much lower than formerly. Indeed, even with the withdrawal for military purposes of approximately 5% of the existing active profession, the balance between supply and demand is probably at least 10 years in the future.

